# Respiratory Epithelial Cells: More Than Just a Physical Barrier to Fungal Infections

**DOI:** 10.3390/jof8060548

**Published:** 2022-05-24

**Authors:** Bianca C. S. C. Barros, Bruna R. Almeida, Debora T. L. Barros, Marcos S. Toledo, Erika Suzuki

**Affiliations:** 1 Laboratory of Applied Toxinology, Center of Toxins, Immune-Response and Cell Signaling (CeTICS), Butantan Institute, São Paulo 05503-900, SP, Brazil; bianca.barros@esib.butantan.gov.br; 2 Department of Microbiology, Immunology, and Parasitology, Escola Paulista de Medicina, Universidade Federal de São Paulo, Ed. Antonio C. M. Paiva, São Paulo 04023-062, SP, Brazil; b.almeida10@unifesp.br (B.R.A.); d.barros@unifesp.br (D.T.L.B.); 3 Department of Biochemistry, Escola Paulista de Medicina, Universidade Federal de São Paulo, Ed. Leal Prado, São Paulo 04023-062, SP, Brazil; ms.toledo@unifesp.br

**Keywords:** epithelial cell, respiratory, lung, airway, *Aspergillus*, *Paracoccidioides*, cytokine, adhesion, invasion

## Abstract

The respiratory epithelium is highly complex, and its composition varies along the conducting airways and alveoli. In addition to their primary function in maintaining the respiratory barrier and lung homeostasis for gas exchange, epithelial cells interact with inhaled pathogens, which can manipulate cell signaling pathways, promoting adhesion to these cells or hosting tissue invasion. Moreover, pathogens (or their products) can induce the secretion of chemokines and cytokines by epithelial cells, and in this way, these host cells communicate with the immune system, modulating host defenses and inflammatory outcomes. This review will focus on the response of respiratory epithelial cells to two human fungal pathogens that cause systemic mycoses: *Aspergillus* and *Paracoccidioides*. Some of the host epithelial cell receptors and signaling pathways, in addition to fungal adhesins or other molecules that are responsible for fungal adhesion, invasion, or induction of cytokine secretion will be addressed in this review.

## 1. Introduction

As we breathe, several microorganisms and other stimuli enter our respiratory system and make the primary contact with the epithelium, which acts not only as a physical barrier but also as an important key for the host’s immune defense. The functional differences and the cell complexity of the epithelia can be verified along the upper respiratory tract (nasal cavity, pharynx, and larynx), through the conducting airways (trachea, bronchi, and bronchioles) and to the alveoli [1,2,3]. Through cell–cell communication, including with immune cells, epithelial cells are essential for respiratory system homeostasis. If the regulatory mechanisms are in disequilibrium, a disease state may be triggered in the individual [2]. 

Histology of the airway epithelium varies according to the region of the respiratory tract. Most of this epithelium is pseudostratified and composed of (i) basal cells, which can renew the epithelium and differentiate into other cell types; (ii) club cells that also have epithelial renewal properties and secrete an important anti-inflammatory protein called uteroglobin; (iii) ciliated cells, which beat their cilia and play an essential role in expelling microorganisms and other foreign particles; (iv) goblet cells, which produce mucus and act with ciliated cells to assist airway clearance; and (v) pulmonary neuroendocrine cells, which are rare cells that secrete neuropeptides, promoting nervous and immune responses (see ref. [2] for review). Tuft cells, pulmonary ionocytes, hillock cells, and microfold cells were also identified in the airways, but the precise functions of these cells in the respiratory system require further study [2]. In contrast, the alveolar epithelium consists mostly of types I and II alveolar epithelial cells (also known as pneumocytes). The type I alveolar epithelial cell is a large and very thin cell, and its primary function is gas exchange. The type II cell has a cuboidal shape and secretory functions, such as surfactant production [3,4].

In addition to forming a physical barrier, lung epithelial cells function as sensors in the respiratory tract, interacting and responding to microorganisms and other particles. Stable lung homeostasis is also maintained by interactions with a sparse and diverse microbiome, but when dysbiosis occurs, lung diseases may develop. Lung microbiome disruption is associated with the progression of chronic obstructive pulmonary disease, cystic fibrosis, and idiopathic pulmonary fibrosis [5,6,7].

Pathogenic bacteria, viruses, or fungi also interact with lung epithelial cells and may exploit host cell receptors, leading to a better cell adhesion (and frequent invasion), establishing an infection in the host. In the context of the host immunological response, epithelial cells present on their surfaces pattern recognition receptors (PRRs) that recognize pathogen-associated molecular patterns (PAMPs) in microorganisms, triggering the release of chemokines and cytokines that modulate the immune response [3]. In fungi, mannans or glucans are examples of PAMPs that are recognized by PRRs such as toll-like receptors (TLRs) and dectins [8].

According to The Global Action For Fungal Infections (GAFFI), 2 million people die of fungal diseases every year [9]. Some of these fungi can cause endemic or opportunistic respiratory mycoses, which can result from the inhalation of fungal spores, reactivation of latent fungi, or hematological dissemination. Endemic fungi are usually found in specific regions of the world, and they are thermally dimorphic, i.e., these fungi exhibit a mycelial form in the environment and a yeast form within the host. While endemic fungi may promote disease in healthy individuals, most opportunistic cases occur in immunocompromised people [10,11].

This review addresses the important responses of epithelial cells to two relevant fungi that cause pulmonary mycoses. The first fungus is the filamentous *Aspergillus* which the COVID-19 pandemic brought to light once again [12], and the second is *Paracoccidioides* which causes an endemic mycosis in Latin America and is considered a neglected disease that may lead to severe sequelae of the lungs and other organs [13].

## 2. *Aspergillus*

Lung diseases caused by *Aspergillus* affect more than 10 million people, resulting in approximately 200,000 deaths annually [14]. This fungus causes a complex variety of diseases that have different pathogenic mechanisms and clinical manifestations, and are associated with patients who exhibit distinct immune responses [15]. Latgé and Chamilos [15] described three major groups of aspergillosis syndromes: (i) hypersensitivity-allergy, which includes severe asthma with fungal sensitization and allergic bronchial pulmonary aspergillosis; (ii) structural disease, including aspergilloma, chronic pulmonary aspergillosis, and semi-invasive forms; and (iii) severe immunodeficiency, which contains invasive pulmonary aspergillosis, invasive bronchial aspergillosis, and central nervous system aspergillosis (extrapulmonary).

Recently, the incidence of COVID-19-associated pulmonary aspergillosis in intensive care units was indicated to be approximately 10% to 15% [16]. This coinfection raises concerns about the high mortality among critically ill patients during the COVID-19 pandemic [12]. The problem requires multiple approaches to find a solution, including prevention, diagnosis, and treatment. In addition, new drugs or treatment protocols are necessary to overcome drug resistance in fungi. As an antifungal agent and an immunoadjuvant, all-*trans* retinoic acid is a promising agent that could be used in combination with classic antifungal drugs, reducing their dose and side effects [17,18].

**Figure 1:**
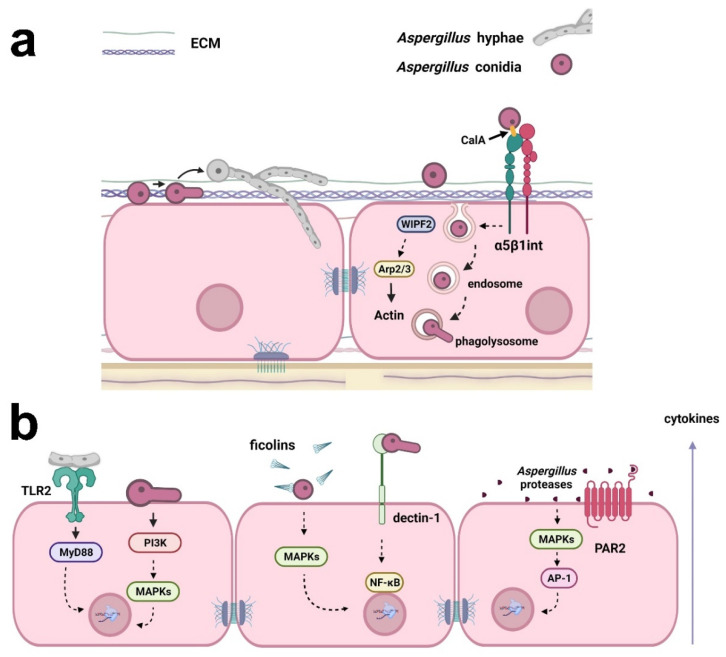
Examples of the responses of respiratory epithelial cells to *Aspergillus* infection. (**a**) *Aspergillus* conidia can bind to extracellular matrix (ECM) proteins and epithelial cell surface. Conidia may germinate on the surface of epithelial cells, and the resulting hyphae can actively invade the cells. Conidia internalization may occur through fungal CalA interaction with host α5β1 integrin and WIPF2, Arp2/3, and actin reorganization. Conidia can also be trafficked in the endosomal system and germinate in phagolysosomes, reentering the extracellular space. (**b**) Cytokine secretion by epithelial cells may be a result of (i) the interaction of *Aspergillus* hyphae with TLR2, activating MyD88; (ii) the activation of PI3K and MAPKs by germinating conidia; (iii) the opsonization of *Aspergillus* conidia by ficolins and MAPK activation; (iv) interaction with dectin-1 and the activation of NF-κB; and (v) activation of MAPKs and the transcription factor AP-1 by *Aspergillus* proteases. *Aspergillus* proteases can also activate PAR2, inducing cytokine production. Created with Biorender.com.

It is essential to understand the signaling processes in host-fungus interactions to pave the way for future clinical improvements, which will benefit the patient. Host-fungus interactions involve a plethora of mechanisms and evoke different explanations for *Aspergillus* invasion in the lung. This is important because lung epithelial cells respond diversely depending on the kind of insult or stress that is causing the injury [19]. The major view on this aspect indicates that conidia can bind and germinate on the surface of alveolar epithelial cells following active hyphal invasion (Figure 1) [20]. Despite the invasion, the morphology of epithelial cells seems to remain unaltered. Whether invasion requires the impairment of cell integrity is still a matter of debate, although there is sufficient evidence suggesting that epithelial invasion by hyphae can be achieved through actin remodeling in bronchial epithelial cells without disturbing morphology or epithelial integrity [21]. Thus, questions remain about the mechanisms responsible for this fungal cell invasion.

Several research groups have been working on identifying the molecules involved in several steps of *Aspergillus* infection. At first, *Aspergillus* conidia adhere mostly to proteins and/or carbohydrates found in the membrane of epithelial cells or the surrounding host extracellular matrix (ECM) [22]. Kerr et al. [23], for example, have indicated that *A. fumigatus* conidia and the fungal lectin FleA, which recognizes fucosylated structures, avidly bind to purified lung mucins. Some proteins that participate in epithelial cell–cell adhesion, such as E-cadherin, were also described as molecules that cooperate in the adhesion of *A. fumigatus* conidia to lung epithelial cells [24]. The polysaccharide galactosaminogalactan, which is found in the hyphal cell wall of *Aspergillus,* mediates adhesion to epithelial cells and fibronectin [25]. Bouchara et al. [26] described that the binding of laminin and fibrinogen to a possible lectin present in *A. fumigatus* conidia was dependent on sialic acid residues present in these glycoproteins, and Warwas et al. [27] indicated that sialidase treatment of *A. fumigatus* spores decreased adherence to fibronectin in coated wells and conidia uptake by lung epithelial A549 cells. Conversely and strangely, the binding of *A. fumigatus* conidia to those cells increased when treated with sialidase. Thus, further studies are necessary to completely understand the role of different carbohydrates, including sialic acids, in fungal adhesion to ECM components or host cells.

In addition to adhesion, *Aspergillus* can also invade respiratory epithelial cells. Liu et al. [28] described that the thaumatin-like protein CalA, which is expressed on the surface of *A. fumigatus* conidia, participates in the mechanism of epithelial cell invasion by interacting with the host receptor α5β1 integrin. When using a CalA mutant, the authors did not observe a reduction of *A. fumigatus* conidia adhesion to the human alveolar epithelial cell line A549, suggesting that although CalA was expressed on the cell surface of the fungus, it was dispensable for the adhesion step. More importantly, endocytosis of the CalA mutant was reduced by epithelial cells, indicating the role of *Aspergillus* CalA as a fungal invasin rather than an adhesin [28]. Another mechanism was described by Culibrk et al. [29], who demonstrated a reduction of conidia internalization by the human airway epithelial cell line 1HAEo- after WAS-interacting protein family member 2 (WIPF2) silencing. As WIPF2 is known to mediate the function of actin related protein (Arp) 2/3 complex, which is responsible for actin reorganization, the authors presented the participation of this protein complex in this process [29].

Some studies have indicated that fungal spores can be absorbed by respiratory epithelial cells efficiently and rapidly, which contributes to *Aspergillus* spores digestion by mature host phagolysosomes [14,30]. Briefly, conidia are internalized and most of them are killed by epithelial cells. The conidia that survive are trafficked by the endosomal system to phagolysosomes, where they germinate and reenter the extracellular space, sustaining infection [22]. NADPH oxidase and reactive oxygen species (ROS) produced by mitochondria are redundant mechanisms by which alveolar macrophages and neutrophils kill fungi [31]. However, human epithelial cells are still neglected in this scenario and few studies have used them as an infection model.

Several studies have assessed the role of immune cells during fungal infection in the respiratory epithelium. For example, it is known that repeated exposure to *Aspergillus* conidia induces neutrophil transmigration [32] through a probable caspase recruitment domain (CARD) 9 signaling-dependent mechanism [33]. A study by Jepsen et al. [34] demonstrated that fibrinogen C domain-containing protein 1 (FIBCD1) acts as a PRR in epithelial cells, recognizing polysaccharides of the *A. fumigatus* cell wall, such as chitin, and modulating the TLR-dependent inflammatory response in the host [34].

A classic response against fungal pathogens involves the expression of interleukin (IL)-8, an important chemokine for neutrophil chemotaxis [35,36]. Liu et al. [37] have indicated that the conidia of *A. fumigatus* can induce IL-8 secretion in a time-dependent manner in A549 epithelial cells. A similar effect was observed when the release of monocyte chemoattractant protein 1 (MCP-1) was analyzed, but not for tumor necrosis factor (TNF)-α [37]. Another study using A549 cells and transcriptome analysis by RNA-seq indicated an increase in IL-6, IL-8, and, in contrast to Liu et al. [37], TNF-ɑ after interaction with *A. fumigatus* conidia. The mRNA level was confirmed by qRT-PCR [38]. This discrepancy between the findings with respect to TNF-ɑ expression in A549 cells may be explained by using different strains of *A. fumigatus* and varying experimental protocols. Meanwhile, the production of IL-6 and IL-8 by epithelial cells due to *A. fumigatus* infection was observed in several studies [39,40,41,42,43,44].

While IL-8 expression by macrophages is a well-known event dependent on TLR activation, the expression of IL-8 by respiratory epithelial cells in response to *Aspergillus* infection may also occur in a myeloid differentiation primary response (MyD) 88-independent manner as indicated by Balloy et al. [41], who demonstrated that germinating conidia promoted IL-8 secretion by epithelial cells after activation of the phosphatidylinositol 3-kinase (PI3K) and mitogen-activated protein kinase (MAPK) pathways [41]. On the other hand, Oya et al. [39] verified that pretreatment with TLR2 blocking antibodies promoted a reduction of IL-6 and IL-8 secretion levels in BEAS-2B bronchial epithelial cells incubated with *A. fumigatus* hyphal fragments, suggesting the involvement of this receptor [39]. In addition, there is evidence that *A. fumigatus* hyphae regulate the expression of IL-1β, IL-1ɑ, and TNF-α by mechanisms that involve the participation of TLR2, but these cytokines are not released by host cells [39]. By using A549 epithelial cells overexpressing FIBCD1, Jepsen et al. [34] verified that this protein suppressed TLR2- and TLR4-dependent IL-8 secretion when cells were infected with the conidia of *A. fumigatus* [34]. In contrast, the overexpression of FIBCD1 increased IL-8 secretion when it was induced by TLR5 in this model [34].

Another receptor that participates in signal transduction during *Aspergillus* infection is the C-type lectin receptor dectin-1 [37,44,45]. Liu et al. [37] demonstrated that *Aspergillus* conidia promoted the secretion of IL-8 and MCP-1 by epithelial cells through dectin-1 and nuclear factor κ-light-chain-enhancer of activated B cells (NFκB) activation [37]. In addition, Sun et al. [40] demonstrated that dectin-1 was regulated by TLR2 and was required for ROS production and TNFα, granulocyte-macrophage colony-stimulating factor (GM-CSF), and IL-8 mRNA expression in human bronchial epithelial cells incubated with *Aspergillus* [40]. 

One of the receptor families that participates in the activation of the lectin complement pathway is the soluble secreted PRR ficolin (in humans, there are three types of ficolins: M, H, and L) [46]. Ficolins have been reported to be mediators of the inflammatory response in epithelial cells due to *Aspergillus* infection by enhancing neutrophil recruitment [47]. H-ficolin, for example, binds to *A. fumigatus* conidia and, after opsonization, induces an increase in IL-8 secretion by A549 epithelial cells. This process is mediated by p38 MAPK, c-Jun N-terminal kinase (JNK), and mitogen-activated protein kinase (MEK) 1/2 [48]. 

Proteases from *Aspergillus* can disrupt epithelial integrity and disturb homeostasis and barrier function, culminating in an inflammatory response and consequently promoting disease in the individual [49,50]. *A. fumigatus* proteases are also described as a potential source for ECM component degradation [50], and most *A. fumigatus* allergens exert proteolytic activity. For example, Asp f 5 is a metalloprotease, while Asp f 13 and Asp f 18 are serine proteases [51,52]. Proteases present in fungi have been proven to interfere with epithelial barrier integrity by disrupting tight junctions, acting on occludin, zonula occludens-1 (ZO-1), and claudins, which increases permeability and facilitates antigen access through the epithelium [53].

Regarding the inflammatory response, Kim et al. [49] verified that *Aspergillus* proteases could increase the expression and secretion levels of IL-1β, IL-6, IL-8, and transforming growth factor (TGF)-β by human primary bronchial epithelial cells. In addition, these authors also demonstrated that proteases from *Aspergillus* induced mitochondrial ROS, which in turn activated MAPK and activator protein 1 (AP-1), enhancing the inflammatory response in these cells [49]. In a recent study, Rowley et al. [44] demonstrated that inhibitors of serine-, cysteine-, and metalloproteases reduced IL-8 level secretion by the human bronchial epithelial 16HBE14o- cell line in the presence of *Aspergillus* conidia, suggesting a potential role of those proteases in the inflammatory response induced by the fungus [44]. Moreover, proteases can cleave an extracellular domain of protease-activated receptors (PARs). PAR2, for example, is a target of fungal serine proteases and participates in IL-33 expression when human sinonasal epithelial cells are incubated with *A. fumigatus* [54].

We know that fungal proteases and extracellular vesicles (EVs) can trigger paracrine signals and prepare the environment for infection while fungal structures are still not in contact with the host tissue. EVs can deliver cargo to tissue, inducing changes in the host and ultimately benefiting the fungus [55,56,57]. On the other hand, although *Aspergillus* EVs can transport proteins related to redox signaling, cell wall remodeling, and lipid/sugar metabolism, they induce M1 macrophage polarization, leading to phagocytosis and fungal clearance [56,58,59]. Whether *Aspergillus* EV interactions with host immune cells are beneficial or detrimental to the fungus remains a matter of debate. Moreover, the role of these EVs when they interact with respiratory epithelial cells remains largely unknown and is an opportunity for further investigation.

Considered together, there is consistent evidence that *A. fumigatus* adheres to the epithelial surface by using a range of receptors and ECM components, activates different receptors, and modulates cell signaling pathways, culminating in inflammatory responses, in addition to affecting epithelial barrier integrity. However, how the initial response in respiratory epithelial cells against *A. fumigatus* drives the immune adaptive response and long-term epithelial remodeling events is still unclear [50].

## 3. *Paracoccidioides*

Paracoccidioidomycosis (PCM) is the most prevalent systemic mycosis in Latin America [60]. It is estimated that 80% of confirmed PCM cases occur in Brazil [61], but until 2020, this mycosis was not considered a mandatory notifiable disease in this country; thus, the true number of individuals affected by PCM is not known. An increase in PCM microepidemics has also been reported in different regions of Brazil. Environmental factors resulting from the opening of new agricultural frontiers through forest clearing may contribute to the current epidemiological situation of this mycosis [62]. Other activities that disturb the soil, such as the construction of highways, as seen in the state of Rio de Janeiro, Brazil [63], can also cause PCM outbreaks. Clusters of this mycosis may also be associated with climate events, such as El Niño, which provide optimal conditions for fungal growth, enhancing human exposure [64]. PCM has a social impact on areas where this disease is endemic because it frequently affects poor rural workers. PCM impacts these individuals by the chronicity of the disease, the long duration of treatment, and sequelae that lead to low quality of life and inability to work [65]. 

The etiologic agent of PCM is the thermally dimorphic fungus *Paracoccidioides,* which comprises the species *P. lutzii* and *P. brasiliensis* [66,67]. While *P. lutzii* mostly occurs in the midwestern and northern regions of Brazil and Ecuador, *P. brasiliensis* is endemic in all Brazilian regions, Argentina, Paraguay, Uruguay, Peru, Colombia, and Venezuela [61,68]. Recently, by analyzing nuclear and mitochondrial genes, Turissini et al. [69] proposed three new species attributed to the cryptic species of *P. brasiliensis* PS2, PS3, and PS4 as, respectively, *P. americana*, *P. restrepiensis*, and *P. venezuelensis* [69,70,71].

It is accepted that infection by *Paracoccidioides* occurs after the inhalation of fungal propagules (mycelium), which are usually present in the soil in rural areas. Once in the host lungs, the mycelium differentiates into yeast. Yeasts may remain in a latent state or trigger the disease [60,70]. The development of PCM is dependent on some characteristics of the host, such as sex, age, and immunological competence, in addition to factors related to the fungus, such as virulence and the ability to modulate the host’s immune system [60]. Some of these characteristics may act as a host protection factor, such as the presence of the female hormone 17-β estradiol, which inhibits the synthesis of proteins involved in the morphological transition from mycelium to yeast, a crucial process for the establishment of the infection, which would explain the prevalence of cases in male patients, even if both sexes are exposed to the same risk factors [72,73,74].

In the last three decades, the interaction and responses of epithelial cells to *Paracoccidioides* infection have been investigated by some groups. One of the first articles that described the interaction of epithelial cells and *Paracoccidioides* yeasts was published in 1994 by Mendes Giannini’s group. Despite the use of Vero cells (monkey kidney) in this work (i.e., not from the respiratory tract), the authors demonstrated for the first time that *Paracoccidioides* yeasts could adhere to these epithelial cells [75]. In the same year, Vicentini et al. [76] indicated that *Paracoccidioides* yeasts were capable of adhering to another epithelial cell (MDCK, canine kidney), and this adhesion was enhanced when laminin was added to the cultures. Interestingly, these authors also observed that laminin increased the number of granulomas when *P. brasiliensis* yeasts were inoculated in hamster testicles, suggesting that laminin was important for *Paracoccidioides* pathogenesis [76]. In fact, following these studies, many *Paracoccidioides* molecules were proven to interact with several ECM proteins. For example, glycoprotein 43, glyceraldehyde-3-phosphate dehydrogenase, and triosephosphate isomerase of *Paracoccidioides* yeasts have been described as capable of binding to laminin and fibronectin [76,77,78,79,80,81,82,83,84,85], while the 19 and 32 kDa proteins, present on the fungal surface, also interact with laminin, fibronectin, and fibrinogen [86]. The 14-3-3 protein, which is widely present in the cytoplasm and fungal cell wall, can also adhere to laminin [87]. Assays performed with alveolar epithelial cells indicated that during infection with *P. brasiliensis* (Pb18 isolate), 14-3-3 protein localized predominantly in the cell wall and antibodies anti-14-3-3 reduced *P. brasiliensis* adhesion, indicating that this protein participates in fungal interaction with host epithelial cells [87].

Studies comparing distinct species of *Paracoccidioides* indicated that fungal adhesion to ECM proteins or epithelial cells differed. Oliveira et al. [88] demonstrated that while *P. brasiliensis* (Pb18 isolate) adhered more to fibronectin than *P. lutzii* (Pb01 isolate), *P. lutzii* presented higher adhesion rates to laminin and collagen types I and IV [88]. Even though these authors verified that the adhesion of these two species to epithelial cells was similar, Almeida et al. [89] demonstrated that *P. lutzii* (Pb01 isolate) adhesion rates were seven times higher than those of *P. brasiliensis* (Pb18 isolate) and *P. americana* (Pb03 isolate) [88,89]. Such differences might be explained by the distinct methods used among the laboratories to analyze fungal adhesion.

Some studies have demonstrated the ability of *P. brasiliensis* yeasts to hijack host epithelial cell signaling to establish fungal infection. Maza et al. [90], for example, observed that *P. brasiliensis* (Pb18 isolate) yeasts promoted clustering of lipid rafts on the surface of alveolar epithelial cells, which is important for fungal adhesion to host cells [90]. Although invasion in epithelial cells is a rare event, Mendes Giannini’s group demonstrated that *P. brasiliensis* (Pb18 isolate) may also invade Vero cells by inducing cytoskeletal rearrangement in the host cell. The same authors also verified that protein tyrosine kinases (PTKs) are involved in the *P. brasiliensis* adhesion and invasion of epithelial cells [91]; moreover, this fungus may promote apoptosis in epithelial cells [92].

**Figure 2:**
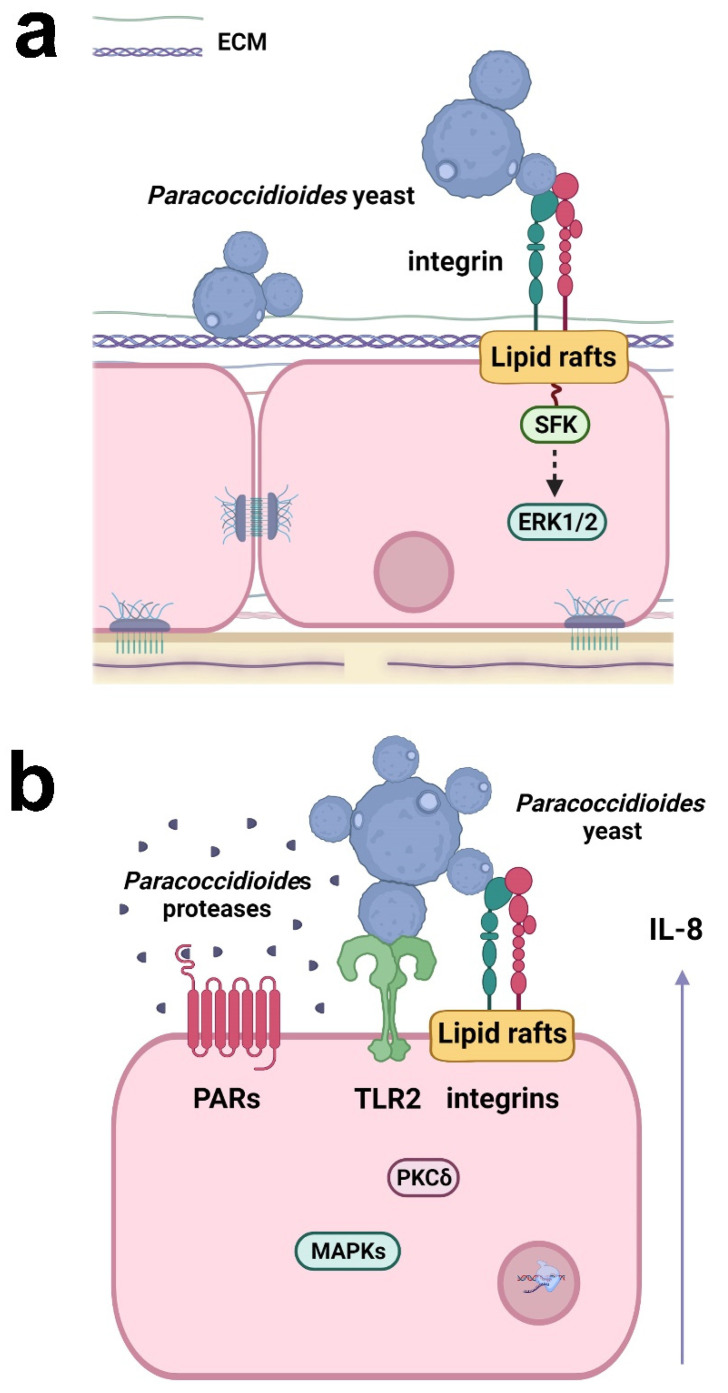
Responses of respiratory epithelial cells to *Paracoccidioides* infection. (**a**) *Paracoccidioides* yeasts can bind to host ECM proteins and α3 and α5 integrins. Fungal adhesion is also dependent on lipid raft clustering, which promotes Src-family kinase (SFK) and ERK1/2 activation. (**b**) *Paracoccidioides* induces IL-8 cytokine secretion in epithelial cells by interacting with α3 and α5 integrins and TLR2. Fungal proteases may also activate PAR-1 and PAR-2 in epithelial cells, leading to IL-8 production. The secretion of IL-8 is promoted by *Paracoccidioides* and is dependent on lipid raft clustering and the activation of MAPK (p38MAPK, ERK1/2) and PKCδ. Created with Biorender.com.

Recently, Almeida et al. [89] demonstrated that α3 and α5 integrins, which are present on the surface of alveolar epithelial cells, participate in the adhesion of *P. lutzii* (Pb01 isolate) (Figure 2). In a previous study, the same group verified that the other species of *Paracoccidioides*, *P. brasiliensis* (Pb18 isolate), promoted an increase in the levels of these integrins in the alveolar epithelial cell line A549 during the first 5 h of infection, but after 24 h, surprisingly, a decrease of α3 integrin levels was observed, which was dependent on direct contact between fungi and epithelial cells [93], indicating that *Paracoccidioides* can manipulate host cell receptor levels along with the infection.

*P. brasiliensis* (Pb18 isolate) can manipulate epithelial cell signaling pathways to secrete the proinflammatory cytokines IL-6 and IL-8, but not the anti-inflammatory cytokine IL-10 [94,95,96]. The interaction of *P. brasiliensis* with epithelial cells promotes the activation of protein kinase C (PKC) δ and the MAPKs p38 and extracellular signal-regulated kinase (ERK) 1/2, which are important for the secretion of IL-6 and IL-8 (Figure 2) [94,95]. TLR2, α3 and α5 integrins, and lipid rafts also participate in IL-8 secretion (Figure 2) [93,96]. Moreover, proteases secreted by *P. restrepiensis* (Pb339 isolate) can also induce cytokine secretion in epithelial cells. Oliveira et al. [97] demonstrated that this fungus secretes serine and cysteine proteases that activate PARs 1 and 2 and then induce IL-6 and IL-8 secretion (Figure 2). 

As observed for the adhesion of *Paracoccidioides* yeasts to alveolar epithelial cells, different species induce distinct cytokine levels in these cells. It was observed that *P. brasiliensis* (Pb18 isolate) promoted the highest IL-8 level secretion, followed by *P. americana* (Pb03 isolate) which induced intermediate levels. *P. lutzii* (Pb01 isolate) promoted the lowest levels of this cytokine. Interestingly, while *P. brasiliensis* and *P. lutzii* promoted IL-8 secretion mostly through secreted products, *P. americana* requires direct contact with the epithelial cell [89], indicating that different species of *Paracoccidioides* induce distinct responses in these epithelial cells.

## 4. Conclusions

Respiratory fungal infectious diseases are widespread around the world and contribute to an increase in the mortality rate among human mycoses. In addition, the usual lack of compulsory notification leads to underestimated epidemiological data, affecting the knowledge of the true number of individuals afflicted with respiratory fungal diseases. In this context, studies that aim to understand the mechanisms behind the establishment of respiratory fungal mycoses are indispensable. In the last decades, several research groups have investigated the responses of respiratory epithelial cells to infections by viruses, bacteria, or fungi. These studies have indicated that epithelial cells act not only as a physical barrier but also as sensors of the respiratory environment, cooperating with the modulation of the host immune response. The literature has indicated several mechanisms by which fungi interact with respiratory epithelial cells, e.g., hijacking of host cell signaling pathways, which leads to fungal adhesion, and, depending on the fungal species, an active invasion into these cells. Moreover, during fungal infection, epithelial cells secrete chemokines and cytokines that mediate communication with host immune cells. Despite the recent progress in understanding the cellular mechanisms of this crosstalk between epithelial cells and immune cells, data available in the literature are still scarce and occasionally contradictory. Thus, more studies are necessary to elucidate the signaling pathways that lead to the establishment of infection and inflammatory responses. A promising strategy consists of in vitro cell culture systems (such as air liquid interface, polymer scaffolds, and organoids—see ref. [98] for review) that are more closely related to the in vivo respiratory system, which may help researchers discover new therapeutic approaches for respiratory infectious diseases.

## Data Availability

Not applicable.
